# Assisted Reproductive Technology in Breast and Gynecologic Malignancies: Cancer Risk, Fertility Preservation, and Post-Treatment Reproductive Outcomes

**DOI:** 10.3390/jcm15155800

**Published:** 2026-07-24

**Authors:** Panagiotis Cherouveim, Esra Cetin, Celine Sooknarine, Shagun Tuli, Bhuchitra Singh, Youssef Youssef, Gaby Moawad, Benedetta Guani, Jean Marc Ayoubi, Anis Feki

**Affiliations:** 1 Department of Obstetrics and Gynecology, Hurley Medical Center, Michigan State University, Flint, MI 48503, USA; 2 Department of Gynecology and Obstetrics, School of Medicine, Johns Hopkins University, Baltimore, MD 21287, USA; 3 Division of Minimally Invasive Gynecology, Department of Obstetrics and Gynecology, Cleveland Clinic, Cleveland, OH 44124, USA; 4 Department of Obstetrics and Gynecology, School of Medicine & Health Sciences, The George Washington University, Washington, DC 20037, USA; 5 Division of Gynecologic Oncology, Department of Obstetrics and Gynecology, Fribourg University Hospital, 1708 Fribourg, Switzerland; 6 Department of Gynecology and Obstetrics and Reproductive Medicine, Hospital Foch, 92150 Paris, France; 7 Department of Obstetrics and Gynecology, Fribourg University Hospital, 1708 Fribourg, Switzerland

**Keywords:** assisted reproductive technology, fertility preservation, gynecologic cancer, reproductive outcomes

## Abstract

Assisted reproductive technology (ART) is increasingly utilized worldwide, yet concerns remain regarding its potential association with breast and gynecological malignancies and the safety of fertility-preservation strategies in cancer survivors. Ovarian stimulation exposes women to supraphysiologic hormone levels, raising questions about cancer risk, particularly for hormone-sensitive tumors. Current evidence, however, is largely reassuring. Registry-based studies and meta-analyses demonstrate no consistent increase in breast or endometrial cancer following ART, although endometrial cancer risk remains inconclusive despite large new cohorts. Cervical cancer has not been linked to ART exposure. For ovarian cancer, risk appears primarily driven by underlying infertility, parity, and endometriosis rather than ART itself, and no excess risk is observed among BRCA mutation carriers. In parallel, fertility preservation (FP) has become an integral component of gynecologic oncology care for reproductive-aged women. Strategies including cryopreservation of oocytes, embryos, or ovarian tissue, as well as fertility-sparing surgery or hormonal therapy, can be safely pursued in carefully selected early-stage cancers. Pregnancy and livebirth rates vary by diagnosis: outcomes are favorable after breast and cervical cancers, more limited in endometrial cancer due to endometrial receptivity challenges, and possible but less predictable in ovarian cancer, where stage and histology guide feasibility. Available observational evidence does not suggest a clear increase in recurrence risk among carefully selected patients, although evidence remains limited and long-term follow-up is needed. Overall, current data suggest ART is safe when individualized to patient and tumor characteristics, highlighting the importance of proactive fertility counseling, modified stimulation protocols, and multidisciplinary care to optimize oncologic and reproductive outcomes.

## 1. Introduction

Infertility prevalence is estimated to be around 12–18% [1]. Despite a variety of procedures being available within the spectrum of Assisted Reproductive Technology (ART) to assist with conception, there remains concern about long-term risks due to scarce evidence, emphasizing the need for comprehensive synthesis identifying the evidence gaps. 

This concern is partially driven by a hypothesized connection of ART with higher risk of cancer [2]. Medications that are often utilized during ART enhance ovarian response and aim at multifollicular growth [3]. This enhanced response often leads to supraphysiologic estradiol levels that peak as high as 5000–10,000 pmol/L [4]. Even though women seeking ART are mostly younger, and their risk for cancer is relatively low [5], gynecological cancers are a common concern as they are often considered hormone-sensitive [6,7]. While this concern relates to a potential link to breast and endometrial cancer, possible relationships to other gynecological cancers such as ovarian or cervical cancer are less clear [8,9,10]. Finally, studying these associations is often challenging because of the protracted duration between exposure to ART and gynecological cancer development and the variety of factors that might also confound this association [11].

Moreover, if women survive the cancer itself, they often receive treatments and undergo procedures that can pose additional physiological and anatomical challenges. These challenges include but are not limited to diminished ovarian reserve caused by chemotherapy agents or irreversible infertility caused by hysterectomy with salpingo-oophorectomy [12,13]. Despite the above-mentioned challenges, these treatment modalities may often be the only option available. On the other hand, depending on a number of patient and treatment factors, one might consider a fertility-sparing option [12,13,14,15], especially for women of childbearing age. The benefit of this approach should be carefully weighed against the potential harm of incomplete treatment with higher recurrence rates.

In routine practice, clinicians are increasingly asked to balance oncologic safety with the desire for future fertility, yet the available evidence is fragmented across cancer types, drug classes, and fertility-preservation strategies. To address this gap, the present review synthesizes current data on ART-related gynecologic cancer risk and post-treatment reproductive outcomes to provide pragmatic counseling guidance for oncologists and reproductive specialists and to highlight key areas where further research is needed. The objective of the study was to review the current evidence evaluating ART utilization with gynecological cancer, including breast, ovarian, endometrial, and cervical cancer, as well as review evidence and recommendations for fertility-sparing treatment options and subsequent fertility outcomes. To avoid conceptual overlap, this review distinguishes between (1) cancer incidence following ART exposure in infertile populations without prior malignancy, and (2) oncologic safety and recurrence risk when ART or fertility-preserving strategies are used after cancer diagnosis and treatment. These questions involve different populations, comparators, and sources of bias, and are therefore discussed separately.

## 2. Methods

We conducted a narrative review using PubMed/MEDLINE (last search: November 2025) with combinations of terms including “assisted reproductive technology,” “ovarian stimulation,” “infertility,” “breast cancer,” “endometrial cancer,” “cervical cancer,” “ovarian cancer,” “fertility preservation,” “oncofertility,” “recurrence,” “live birth,” and “pregnancy outcomes.” We also performed backward and forward citation screening of key reviews and included studies to identify additional references (“snowballing”). Inclusion criteria included human studies in English reporting on cancer incidence or recurrence after ART or fertility drugs, and/or reproductive outcomes after fertility-preserving management of gynecologic (and breast) cancers. Studies were selected based on relevance to ART-related cancer incidence, FP, oncologic safety, and reproductive outcomes after cancer treatment. Discrepancies regarding study inclusion were resolved through discussion and consensus among the authors. This was a narrative (non-systematic) review intended to provide a clinically oriented synthesis of heterogeneous literature; formal PRISMA screening, study-level risk-of-bias assessment, and quantitative meta-analysis were therefore not performed.

We prioritized large population-based cohorts/registries, meta-analyses, and well-designed comparative studies; case series were considered when higher-level evidence was lacking (e.g., rare histologies or fertility-sparing procedures). Data were abstracted on population characteristics (including infertility diagnosis, parity, endometriosis, and BRCA status when available), exposure (ART modality, stimulation protocols), and outcomes (cancer incidence/recurrence; clinical pregnancy and live birth). 

Given heterogeneity in designs and effect measures, findings were synthesized qualitatively without meta-analysis. Most included studies were published between 1995 and 2025, reflecting the evolution of ART practices and contemporary oncologic management. Grey literature and conference abstracts were not systematically included, with emphasis placed on peer-reviewed full-text publications.

## 3. Cancer Incidence After ART in Infertile Populations

A. Breast cancer

(i) Invasive vs. in situ breast cancer incidence following ART

Breast cancer risk has long been found to be associated with estrogen and progesterone exposure [16,17]. Although ovarian stimulation might affect breast density [18], and complicate breast cancer diagnosis, evidence on breast cancer risk following ART is largely inconclusive, with most of the studies having a limited follow-up period. 

A previous meta-analysis from 2014 concluded that there was no significant association between ART and breast cancer [19]. Similarly, a recent cohort study of 25,108 women with a more extensive follow-up period (median: 21.1 years) did not find a significantly increased breast cancer risk in women undergoing ART [20]. Machtinger et al. [21] followed 25,108 women who underwent ART and found no increased risk of breast cancer compared with the general population after a mean follow-up of 9.1 years. Similarly, Reigstad et al. [2] studied 19,158 women exposed to ART in Norway and reported no increased breast cancer risk during a mean follow-up of 11 years. In the latter, however, there was an elevated breast cancer risk among women with a prior livebirth that were stimulated with clomiphene citrate during their ART cycles, without a dose-dependent relationship being present [2]. In the general population and among infertile women undergoing ART, the majority of large cohort studies and meta-analyses do not demonstrate a clinically relevant increase in breast cancer incidence following ovarian stimulation, age at ART exposure appears to modify risk estimates in some studies [22]. Although, there have been significant differences between groups in factors that might have confounded the observed differences, including marital, immigration, and educational status. Another large cohort study including 255,786 women treated with ART in Great Britain, with 2.2 million person-years of follow-up, found a higher risk of in situ breast cancer, with risk increasing in relation to the number of ART cycles. However, there was no significant association with invasive breast cancer [9]. On the contrary, the number of previous ART cycles was also associated with decreased breast cancer risk [20]. Some studies reporting lower breast cancer incidence among ART-treated women have suggested potential healthy-user bias, differences in reproductive patterns, or residual confounding rather than a true protective effect of ART itself [9,20]. 

(ii) Breast cancer in BRCA mutation carriers

With respect to hereditary cancer risk, available cohort data do not demonstrate a clear increase in the incidence of primary breast cancer following ovarian stimulation or IVF in women carrying BRCA1 or BRCA2 mutations. While one study suggested a possible increased risk among very young BRCA1 carriers exposed to ART, other cohort studies have not observed an overall increase in breast cancer risk among BRCA mutation carriers. However, sample sizes remain limited and follow-up duration varies, underscoring the importance of long-term surveillance in this high-risk population [23]. 

In summary, current evidence does not demonstrate a consistent association between ART and breast cancer incidence. However, because available data are predominantly observational and remain susceptible to residual confounding, these findings should not be interpreted as definitive proof of oncologic safety. Due to potential diagnostic challenges posed by higher breast density [18], careful consistent breast cancer screening should be considered. 

B. Endometrial cancer incidence following ART exposure

Endometrial carcinoma is another hormone-sensitive gynecological cancer [11]. In spite of ART medication leading to higher estradiol levels having already been established [3], there has been no clear evidence for or against the correlation of ART medication with endometrial cancer. 

There have been many studies that have found no association of ART with endometrial cancer regardless of the medication that was utilized for ovarian stimulation purposes [9,24,25,26,27,28,29,30,31]. Notably, the most recent long-term Dutch cohort with a median follow-up of approximately 24 years did not demonstrate a dose–response relationship with increasing number of ART cycles or longer duration of follow-up, and suggested that observed endometrial cancer risk is largely attributable to underlying patient factors such as obesity, endometriosis, and nulliparity, rather than ART exposure itself [24]. On the other hand, there has been some evidence associating ovarian stimulation agents in general [10,32,33] or specific medications, such as clomiphene [2,34,35], with endometrial cancer risk. The largest and most recent studies, however, report inconsistent results [2,9,10]. In the UK, Williams et al. [9] examined 255,786 women treated with ART contributing 2.2 million person-years of follow-up (mean 8.8 years) and found no significant increase in endometrial cancer risk compared with the general population (RR 1.09, 95% CI 0.90–1.30). In contrast, Kessous et al. [10] studied 106,013 women who underwent fertility treatments in Israel with a mean follow-up of 12 years and reported a significantly higher risk of endometrial cancer (RR 1.56, 95% CI 1.17–2.08). 

Finally, in the Norwegian registry-based cohort of 19,158 women exposed to ART, Reigstad et al. [2] found that use of clomiphene citrate was associated with a higher risk of endometrial cancer, particularly among nulliparous women (RR 2.49, 95% CI 1.43–4.33) and in parous women after ≥6 ART cycles (RR 2.05, 95% CI 1.20–3.52). Importantly, signals suggesting an increased risk of endometrial cancer with clomiphene citrate are derived primarily from older studies involving high cumulative doses and prolonged exposure, often in ovulation induction protocols that predate contemporary ART practices, limiting their applicability to current treatment regimens. Proposed mechanisms include prolonged estrogen receptor stimulation resulting from clomiphene’s long half-life, repeated ovulatory stimulation, and confounding by underlying infertility diagnoses associated with increased baseline cancer risk [24,36]. However, evidence remains inconsistent and causality has not been established.

In conclusion, despite endometrial cancer being hormone-sensitive, current evidence remains inconsistent and does not demonstrate a clear association with ART exposure [2,9,10,24,25,26,27,28,29,30,31,32,33,34,35]. The conflicting findings likely reflect differences in study populations, comparator groups, infertility characteristics, duration of follow-up, and cumulative treatment exposure rather than definitive evidence for or against a causal relationship.

C. Cervical cancer

Cervical cancer is not classically considered to be a hormone-sensitive disease. However, cervical cancer has been associated with parity [37] and hormonal changes in the human papilloma virus (HPV) microenvironment might interfere with the natural history of the HPV infection and its progression to cervical cancer [38]. 

A meta-analysis by Siristatidis et al. [39] shows no increased risk for cervical cancer following ART. Results were similar regardless of whether the general population [2,27,40,41] or women with infertility were utilized as a reference group [27,40,41,42,43]. One of the largest and most recent studies based on a Norwegian registry found no significant association with ART utilization [2]. 

To summarize, current evidence suggests that cervical cancer is not associated with ART utilization. Importantly, this lack of association has been consistently observed across studies using both general-population comparators and infertile control groups, highlighting that HPV vaccination and routine cervical cancer screening remain the primary preventive strategies, independent of ART exposure.

D. Ovarian cancer

(i) Invasive epithelial ovarian cancer risk after ART

Ovarian cancer has several well-established risk factors and the lowest survival rate amongst gynecological cancers [11]. Initial attempts to investigate the potential association of ovarian cancer with ART exposure have produced conflicting results, with some of the studies reporting a significant association [44,45] while others reported none [29,33,46,47]. The potential association in these studies was significantly confounded by factors like parity and infertility diagnosis, among others. More recent studies have suggested that the association between ART and ovarian cancer may be influenced by underlying infertility [48], particularly in women with endometriosis [8,9,22]. 

(ii) Endometriosis-associated ovarian cancer 

In the large Danish population-based study by Vassard et al. [22], which included 58,534 women treated with ART and followed for a median of 9.6 years, 231 cases of ovarian cancer were observed, corresponding to an overall risk similar to the general population (RR 1.02, 95% CI 0.87–1.19), although women with endometriosis had a significantly higher risk (RR 1.79, 95% CI 1.20–2.66). Likewise, Stewart et al. [8] analyzed 21,026 patients undergoing ART in Western Australia and found that the increased risk was confined to women with endometriosis (RR 2.46, 95% CI 1.19–4.92). 

(iii) Borderline ovarian tumors (BOT)

Importantly, recent large population-based cohorts [9,48] consistently distinguish between invasive epithelial ovarian cancer and BOT, showing inconclusive results in invasive ovarian cancer risk after ART, while reporting a modest but reproducible increase in BOT, particularly in younger women and those undergoing repeated treatment cycles. Williams et al. [9], in a cohort of 255,786 women treated with ART in Great Britain contributing 2.2 million person-years of follow-up, reported no significant association with invasive ovarian cancer (RR 0.98, 95% CI 0.87–1.10) but a higher risk of BOT (RR 1.54, 95% CI 1.30–1.81). Similarly, Spaan et al. [48] studied 30,625 ART patients in the Netherlands over a median follow-up of 24 years and found increased risks of both invasive ovarian cancer (RR 1.43, 95% CI 1.20–1.70) and borderline tumors (RR 1.83, 95% CI 1.36–2.46). BOT should be considered separately from invasive epithelial ovarian cancers because they differ substantially in biological behavior, recurrence patterns, fertility outcomes, and prognosis.

(iv) Detection bias and surveillance effects

Finally, detection bias has been proposed as a partial explanation for the excess risk observed within the first 1–2 years after ART initiation, reflecting more intensive pelvic imaging and clinical surveillance during fertility treatment, rather than an immediate carcinogenic effect of ovarian stimulation. In the Danish cohort by Vassard et al. [22], the elevated risk was limited to the first 2 years after ART, coinciding with periods of more intensive sonographic surveillance. 

(v) BRCA-associated hereditary ovarian cancer

There was no significantly higher risk of ovarian cancer following ART even among women with BRCA1 or BRCA2 mutations. In a multicenter cohort study of 1073 BRCA1/2 carriers, including 164 women exposed to fertility treatment, Gronwald et al. [49] found no increased risk compared with carriers who had not undergone ART (RR 0.63, 95% CI 0.21–1.90). This suggests that ovarian stimulation for ART does not further elevate baseline cancer susceptibility conferred by hereditary syndromes. Given that women with BRCA mutations already face a substantially increased lifetime risk of ovarian and breast cancers, the safety of FP and ART in this population is of particular clinical relevance. From a practical standpoint, these data support offering ART to BRCA1/2 mutation carriers when clinically indicated, with careful attention to timing and a strong emphasis on completion of risk-reducing salpingo-oophorectomy once childbearing is complete. For BRCA1 carriers, FP should ideally be completed before recommended risk-reducing salpingo-oophorectomy, generally between ages 35–40 years, whereas BRCA2 carriers may defer surgery until ages 40–45 years. Patients should also be counseled regarding the availability of preimplantation genetic testing for monogenic disease (PGT-M) to reduce transmission of pathogenic variants.

In summary, current evidence indicates that ovarian stimulation protocols, even those requiring supraphysiologic gonadotropin exposure, do not appear to accelerate carcinogenesis in this high-risk group [22,49]. Nevertheless, careful individualized counseling, consideration of alternative FP strategies, and close oncologic surveillance remain important when ART is pursued in women with hereditary cancer syndromes. 

Taken together, the available evidence does not demonstrate a consistent association between ART exposure and invasive epithelial ovarian cancer. The heterogeneity observed across studies likely reflects differences in comparator groups, underlying infertility diagnoses, parity, prevalence of endometriosis, duration of follow-up, cumulative ovarian stimulation exposure, and the extent of adjustment for these confounding factors [9,22,50]. In contrast, the modest increase in BOT incidence reported in several contemporary cohorts may also be influenced by surveillance bias, as women undergoing ART are more likely to receive repeated pelvic imaging and clinical evaluation, increasing the detection of indolent lesions. Consequently, the currently observed associations appear more consistent with differences in patient characteristics and study methodology than with a direct carcinogenic effect of ART itself. Nevertheless, because the available evidence remains predominantly observational, these findings should be interpreted as the absence of a demonstrated association rather than definitive proof of oncologic safety.

## 4. Use of ART After Cancer Treatment: Oncologic Safety and Reproductive Outcomes

A. Breast cancer

(i) FP before breast cancer treatment

Breast cancer treatment rarely involves surgical removal of the pelvic reproductive organs (Table 1). However, breast cancer treatment might involve systemic therapy, including chemotherapy (CT) and endocrine therapy (ET). Therefore, according to the European Society for Medical Oncology (ESMO), it is crucial for women to be counseled regarding the risks of the aforementioned treatments, including amenorrhea and premature menopause, among others, often necessitating pre-treatment counselling with fertility specialists [12]. Oncofertility programs and services were noted to increase the access to and counseling on FP [51,52]. They also had positive outcomes on psychosocial factors including relationships, coping mechanisms and post-traumatic stress [53].

An additional consideration is the prolonged duration of adjuvant ET. Many premenopausal women with hormone receptor (HR)-positive breast cancer receive tamoxifen for 5–10 years, meaning that by the time endocrine therapy is completed, patients are several years older with expected age-related declines in ovarian reserve and natural fertility [54]. Early FP with oocyte or embryo cryopreservation allows gametes to be banked at a younger age and may also shorten the necessary interruption of tamoxifen when attempting pregnancy, since embryos or oocytes are already available rather than requiring a full cycle of ovarian stimulation off therapy [55,56]. Contemporary reviews similarly emphasize that early cryopreservation is a critical strategy for mitigating the dual challenges of aging and prolonged endocrine therapy in young breast cancer survivors [57].

Additionally, there is a variety of FP options for women undergoing treatment for breast cancer, such as embryo, mature oocyte, or ovarian tissue cryopreservation [58]. There is also the option of natural cycle in vitro fertilization (IVF) or gonadotropin-releasing hormone (GnRH) agonist treatment [59]. Each of these options has its own advantages and disadvantages. Selection should carefully weigh patient preferences, availability of treatment modalities, financial burden, and patient compliance with modalities that require frequent administration for long periods of time [12]. American Society of Clinical Oncology (ASCO) [60] and ESMO [12,61] currently recommend GnRH agonists to reduce the impact that CT and ET have on ovarian function and reduce the risk of premature ovarian insufficiency, even for women with a prior history of HR-positive tumor [61]. Nonetheless, patients should be counseled for potential short-term side effects when GnRH is added to chemotherapy, as suggested by the OPTION trial [62]. 

(ii) ART after breast cancer treatment

There is limited data for women attempting conception following breast cancer treatments, and they often pursue fertility treatments to achieve pregnancy, utilization of which increased over time [63] (Table 2 and Table 3). Dieci et al. [64] stressed the significance of addressing fertility issues in breast cancer patients. In their study population of 590 patients less than 40 years old, discussion of fertility issues varied from 58.1% for older patients (>35 years old) in the early years of the study period to 80.7% for reproductive-aged patients in the later years of the study period. There were 38 spontaneously conceived pregnancies from 26 patients after a median of 64 months (19–108 months range) after breast cancer diagnosis. In a cohort of 153 patients with stage I-III breast cancer, overall, 31 (20.3%) achieved pregnancy [21 (67.7%) had previously undergone FP, while 10 (32.3%) did not] [65]. Authors concluded that prior FP treatment increased patients’ chances of at least one conception, while there were no differences in spontaneous abortion rates [65]. Another smaller study showed that 62% of patients were able to conceive naturally after breast cancer treatment, with 38% using ART, 74% of which tried natural conception initially [66]. The POSITIVE trial primarily evaluated the safety of temporary interruption of adjuvant endocrine therapy to allow pregnancy attempts in women with early-stage HR-positive breast cancer. Within this context, available data have not identified a short-term increase in recurrence risk among carefully selected patients who temporarily interrupted endocrine therapy to pursue pregnancy. However, follow-up remains limited, and long-term oncologic safety requires continued surveillance [67]. Even though the utilization of autologous vs. donor oocytes seems to be the main determinant of outcomes, breast cancer survivors have the lowest livebirth rates among cancer survivors (estimated to be around 14.3%) [68]. Of those who were able to conceive, the rates of childbirth were higher in those who had used ART [69]. 

(iii) Recurrence risk after pregnancy and definitive treatment

Available evidence is generally reassuring that pregnancy after breast cancer treatment does not increase recurrence risk, including among patients with HR-positive disease, although counseling should be individualized based on tumor biology, stage, nodal status, time from diagnosis, and need for endocrine therapy. In the POSITIVE trial, temporary interruption of adjuvant endocrine therapy to attempt pregnancy did not appear to worsen short-term breast cancer outcomes, though longer follow-up remains important [67]. Similarly, systematic reviews and meta-analyses have not demonstrated worse disease-free survival or overall survival among women who conceive after breast cancer [73,74]. After definitive surgery, including mastectomy or breast-conserving surgery with appropriate systemic therapy, pregnancy is not considered contraindicated once oncologic treatment is complete and the patient has been counseled regarding baseline recurrence risk and timing of conception. 

Taken together, these findings highlight both the feasibility and safety of FP in breast cancer patients, while underscoring the continued need for proactive counseling, individualized treatment planning, and long-term follow-up to optimize reproductive and oncologic outcomes.

Practical considerations for FP in breast cancer include:

- Timing: Oocyte or embryo cryopreservation should be discussed as early as possible after diagnosis, ideally before initiation of chemotherapy and prior to prolonged endocrine therapy.
- Stimulation protocols: In HR-positive disease, letrozole-based ovarian stimulation protocols (with or without tamoxifen) are commonly used to limit estrogen exposure while achieving adequate oocyte yield.
- Reproductive outcomes: Breast cancer survivors generally experience lower live birth rates compared with survivors of other malignancies, reflecting treatment-related gonadotoxicity and delayed childbearing; however, ART substantially improves the likelihood of achieving pregnancy and live birth among those who attempt conception.

B. Endometrial cancer

(i) Fertility-sparing treatment for atypical hyperplasia/endometrioid intraepithelial neoplasia (AH/EIN) and grade 1 endometrial cancer

A fertility-preservation approach in endometrial cancer is rarely adopted. According to ESMO [15], fertility-preservation should only be considered for patients with AH/EIN or grade 1 endometrioid endometrial carcinoma without myometrial invasion or genetic risk factors (Table 1). This option should only be offered after extensive counselling. Fertility-sparing treatments are not considered the standard of treatment for endometrial cancer [15]. There are several treatment options that can be offered in cases when FP is strongly desired, including progestin therapy (medroxyprogesterone acetate or megestrol acetate) with or without prior hysteroscopic resection [75,76]. Another alternative would be progestins with a levonorgestrel intrauterine device (LNG-IUD), with or without GnRH analogs [15]. One retrospective study showed that LNG-IUD for conservative management of atypical hyperplasia and early-stage endometrial cancer had improved ART outcomes [77]. The role of LNG-IUD-based therapy has become increasingly recognized, often in combination with oral progestins or metformin, with recent multicenter cohorts reporting encouraging pregnancy and live birth outcomes, supporting early referral for ART once complete remission is achieved [78]. Increasing evidence suggests that combined hysteroscopic tumor resection followed by progestin therapy is associated with higher complete response and pregnancy rates compared with systemic progestin therapy alone, likely reflecting improved local disease control and endometrial assessment [79]. Conservative management mandates strict surveillance, including regular clinical assessment and transvaginal ultrasound, together with scheduled endometrial histologic reassessment, typically at 3–6-month intervals. Hysteroscopic-guided endometrial biopsy is generally preferred over blind sampling to accurately assess treatment response and detect persistence or recurrence. 

Emerging molecular classification systems may further refine patient selection for fertility-sparing treatment. Patients with DNA polymerase epsilon catalytic subunit (POLE)-mutated tumors appear to have particularly favorable prognoses and excellent oncologic outcomes, making them potential candidates for conservative management in carefully selected cases [80,81]. Conversely, p53-abnormal tumors are associated with aggressive biological behavior, higher risk of recurrence, and poorer survival outcomes and are therefore generally considered poor candidates for fertility-sparing treatment [80,82]. Data regarding MMR-deficient (MMRd) tumors remain limited, although emerging evidence suggests lower complete response rates and higher recurrence rates following progestin-based therapy compared with MMR-proficient tumors [83,84]. Consequently, further prospective studies are needed before molecular classification can be fully integrated into fertility-preservation algorithms and treatment decision-making [80,85]. Finally, due to high chance of recurrence, hysterectomy and bilateral salpingo-oophorectomy are recommended after family building goals are met [15].

(ii) Pregnancy outcomes and ART after fertility-sparing treatment

Even in cases where every attempt is made to preserve fertility in patients with endometrial cancer, clinical pregnancy and livebirth rates remain suboptimal [70,86] (Table 2 and Table 3). This is no surprise as the endometrium, the main site of implantation and maternal-fetal interaction, is affected. Patients should be extensively counselled and educated about these outcomes, especially those of advanced reproductive age (age > 35 years). Even though there is inconclusive evidence on whether ART improves pregnancy and livebirth rates, broader use of ART should be encouraged, and a fertility specialist should be counseled once clinical response is achieved to optimize fertility outcomes [70,87,88,89]. Pregnancy rates varied from 18.3% to 61.0%, with older age, nulliparity, and need for re-treatment being negative prognostic factors [70,89,90,91,92,93,94]. Live birth rates also varied from 13.3% to 45.0%, with similar negative prognostic factors [89,90,91,94,95]. Spontaneous abortion and ectopic pregnancy rates were similar to the rates observed for the general population (approximately 20.0% and 2.0%, respectively) [70,96]. Furthermore, there were no significant differences in the antepartum period noted between ART-related pregnancies and spontaneous pregnancies [78]. In conclusion, in carefully selected and closely monitored patients, typical pregnancy rates are approximately one-third to one-half, with live birth rates around one-quarter to one-third, although outcomes are strongly influenced by patient age, duration of conservative management, and need for retreatment [78].

C. Cervical cancer

According to the European Society of Gynaecological Oncology (ESGO), there are certain requirements that need to be met in order to consider fertility-sparing treatment for cervical cancer [14] (Table 1). A treatment approach preserving fertility should be offered only for squamous cell carcinomas or usual-type adenocarcinoma (related to HPV) with a maximum diameter of 2 cm, only after extensive patient counseling. FP should not be offered in cases of other histological subtypes (e.g., neuroendocrine tumors and adenocarcinomas not related to HPV) [14]. Even though there is no guarantee, ultrasonography and pelvic MRI are often used to determine the extent of the lesion, and patients should be counseled about the need for a more radical approach in case a more extensive lesion is detected intraoperatively. Another important consideration is the need for a cesarean delivery due to the permanent cervical cerclage placement after simple or radical trachelectomy [14]. 

There is heterogeneity in the literature about pregnancy outcomes following FP in cases of cervical cancer, potentially reflecting the spectrum of operative approaches for cervical cancer treatment [cervical conization (simple and repeated) and trachelectomy (simple and radical, utilizing a vaginal or abdominal approach)] [96,97,98] (Table 2 and Table 3). There have been several factors implicated in these treatment modalities that could be predictive of pregnancy outcomes, with the volume of the cervical tissue that is removed being most crucial [99]. Clinical pregnancy rates were higher following simple compared to radical trachelectomy (65.0% vs. 53.6%, respectively) [99]. Moreover, there is variation in pregnancy rates depending on the approach of radical trachelectomy (67.5% for vaginal vs. 41.9% for abdominal) [71]. Similar variations were observed for livebirth rates (86.4% for simple, 63.4% for vaginal radical, and 41.9% for abdominal radical trachelectomy) [71]. It is also noteworthy that as many as 20% of these pregnancies were conceived following ART utilization [99]. 

Patients should also be counselled about the potential complication of cervical stenosis, the incidence of which is affected by cervical cerclage placement, anti-stenosis tools utilization, and length of the uterus postoperatively [100,101]. Cervical stenosis is detected in approximately 5–10% of cases of cervical cancers that underwent fertility-sparing treatment [102,103]. Assisted reproductive technologies can partly overcome cervical stenosis and altered cervical mucus following trachelectomy; however, implantation challenges and obstetric risks, including preterm birth and preterm premature rupture of membranes, remain elevated compared with the general obstetric population. From a practical standpoint, early referral for ART should be considered in women with postoperative cervical stenosis or recurrent early pregnancy loss, as these patients often benefit from IVF with transcervical or transmyometrial embryo transfer performed in specialized centers.

D. Ovarian cancer

Ovarian cancer is usually first diagnosed in an advanced stage, precluding the adoption of a FP approach (unilateral salpingo-oophorectomy with complete surgical staging) [13] (Table 1). From a clinical and prognostic standpoint, it is essential to distinguish between stage I invasive epithelial ovarian cancers and BOT, as their long-term oncologic behavior, recurrence risk, and reproductive outcomes following fertility-sparing surgery differ substantially. It is considered safe to manage cases of low-grade serous, endometrioid, or mucinous expansile histological subtypes FIGO stage IA by preserving fertility [104,105,106]. On the other hand, management of stage IC tumors becomes more complicated. FP might be a more acceptable approach for stage IC1; however, higher stages (IC2, IC3, and grade 3) have been associated with higher recurrence rates and are more controversial, requiring extensive counseling [107]. FP is contraindicated in stages II and III tumors, due to high risk of recurrence, despite not having clear evidence of the etiology (natural disease history vs. surgical approach) [13,104]. 

Pregnancy rates reported following FP treatment for ovarian cancer depend highly on disease stage and follow-up periods (Table 2 and Table 3). In women with BOT, fertility-sparing surgery followed by spontaneous conception or ART can achieve high cumulative pregnancy rates, often exceeding 60–70% in younger patients with preserved ovarian reserve, albeit at the cost of an increased but generally manageable risk of recurrence, which is typically amenable to repeat surgical management [108]. Overall conception rates vary from 23% in studies with short-term follow-up to 79.0% in studies with longer follow-up (up to 15 years) [109,110,111,112]. Livebirths also varied according to the follow-up period from 20.0% to 73.0% [109,112]. ART utilization rates varied between 4.0% and 30.0% [109,110,111,113,114,115,116,117]. ART utilization was also one of the main determinants of successful conception (Pooled pregnancy rate: 80.0% with ART vs. 54.0% without ART) [72]. Other contributing factors to conception were type of surgical treatment (best fertility outcomes following cystectomy), patient age, and ovarian reserve (median age: 36 years among those who conceived vs. 45 years among those who did not) [115,118,119,120]. Healthcare providers should also take into account the theoretical risk of malignant cell reimplantation in cases of ovarian tissue cryopreservation in this population, an approach requiring careful patient counseling. From a practical perspective, the choice between cystectomy and unilateral salpingo-oophorectomy should balance recurrence risk against preservation of ovarian reserve, with cystectomy offering superior fertility potential but a higher recurrence risk, particularly in cases of bilateral borderline disease-necessitating individualized counseling and long-term follow-up.

### Strengths and Limitations of Current Evidence

The current evidence base is dominated by observational cohort studies, registry-based analyses, retrospective series, and a limited number of prospective studies. While several investigations include large populations and long-term follow-up, important methodological limitations remain (Table 2).

Residual confounding remains one of the greatest limitations of the available literature because infertility itself is associated with several established cancer risk factors, including nulliparity, obesity, polycystic ovary syndrome, endometriosis, and inherited cancer susceptibility. Consequently, it is often difficult to separate the effects of ART from the underlying characteristics of the treated population. In addition, comparator groups differ substantially across studies. Some investigations compare ART-treated women with the general population, whereas others use infertile women who did not undergo ART. These comparator populations differ in their baseline cancer risk, reproductive history, and prevalence of infertility-related conditions, contributing to inconsistent estimates across studies.

Heterogeneity is particularly evident for endometrial cancer and borderline ovarian tumors. Differences in study design, duration of follow-up, cumulative exposure to ovarian stimulation, infertility diagnosis, adjustment for parity and obesity, and inclusion of older versus contemporary stimulation protocols likely contribute to the conflicting findings reported across cohorts. Similarly, the modest increase in borderline ovarian tumor incidence observed in several studies may partly reflect surveillance bias, as women undergoing ART frequently receive repeated pelvic imaging and clinical follow-up, increasing the likelihood of detecting indolent lesions that might otherwise remain undiagnosed. These methodological differences should be considered when interpreting apparent discrepancies between studies rather than assuming true biological differences in ART-related cancer risk.

Surveillance bias may also influence observed associations, particularly for ovarian cancer and BOT, as women undergoing fertility treatment often receive more frequent pelvic imaging and clinical follow-up. Furthermore, ART protocols have evolved considerably over the past three decades; therefore, findings from older cohorts may not fully reflect outcomes associated with contemporary antagonist protocols, random-start stimulation, DuoStim approaches, or letrozole-based stimulation regimens.

Evidence is strongest regarding breast and cervical cancer, where multiple large cohort studies consistently demonstrate no meaningful increase in cancer incidence following ART. Greater uncertainty remains regarding endometrial cancer and BOT, where findings remain inconsistent and longer-term prospective data are needed.

## 5. Conclusions

Available evidence from large registry studies and long-term cohort data does not demonstrate a consistent association between ART and adverse oncologic outcomes among survivors of breast and gynecologic malignancies. For ovarian cancer, the available data suggest that observed associations are more likely attributable to underlying patient characteristics, including infertility, nulliparity, and endometriosis, rather than ART exposure itself. Similarly, current evidence has not demonstrated that ART increases recurrence risk in hormone-sensitive breast or endometrial malignancies, nor has an association been established between ART and cervical cancer development or recurrence. However, because the available evidence is derived predominantly from observational studies, these findings should be interpreted as the absence of a demonstrated association rather than definitive proof of oncologic safety.

In carefully selected patients with breast, endometrial, cervical, and early-stage ovarian cancers, fertility preservation and subsequent ART appear to be feasible without a clear increase in oncologic risk, although evidence remains largely observational and long-term prospective data are limited. Importantly, many studies included in this review evaluated women treated with ART protocols that differ substantially from contemporary practice. Nevertheless, robust long-term data evaluating the oncologic outcomes of these modern protocols remain limited, underscoring the need for continued prospective investigation.

Clinically, fertility counseling should begin at the time of cancer diagnosis to maximize opportunities for FP prior to gonadotoxic treatment or surgical removal of reproductive organs. Ultimately, optimizing both oncologic safety and reproductive outcomes requires early multidisciplinary collaboration among gynecologic oncologists, reproductive endocrinologists, medical oncologists, and obstetric teams, with individualized decision-making regarding ART timing and long-term surveillance.

Future research should prioritize prospective multicenter studies evaluating modern ART protocols, long-term oncologic outcomes, and standardized adjustment for key confounding factors. Importantly, while current evidence is broadly reassuring, the absence of a demonstrated association should not be interpreted as definitive proof of oncologic safety, particularly for hormone-sensitive and ovarian malignancies.

## Figures and Tables

**Table 1:** Practical counseling summary: fertility-sparing eligibility, FP options, ART timing, and obstetric risks (by cancer type).

Cancer Type	Oncologic Eligibility for Fertility-Sparing/FP (Typical Candidates)	Preferred FP Options	When ART Can Be Considered	Key Obstetric Risks/Counseling Points
**Breast cancer** (early-stage, HR+ emphasized)	Not usually requiring pelvic organ removal. Key is systemic therapy impact; pregnancy planning in HR+ depends on endocrine therapy (ET) duration and oncologic risk.	Embryo/oocyte cryopreservation prior to chemotherapy/ET; GnRH agonist during chemo for ovarian function preservation (adjunct); consider letrozole-based stimulation protocols (institution-dependent).	After completion of acute therapy and when deemed safe by oncology; for HR+ often after a planned, supervised temporary ET interruption for pregnancy attempt; ART/embryo transfer can be used during the pregnancy attempt window.	Timing around ET interruption; limited long-term recurrence follow-up for some strategies; pregnancy generally feasible; emphasize multidisciplinary plan (oncology + REI).
**Endometrial** (AH/EIN or grade 1 endometrioid EC)	AH/EIN or FIGO IA, grade 1 endometrioid EC, no myometrial invasion on MRI/TVUS; no contraindicating molecular/genetic risk factors; highly motivated and compliant with surveillance.	Progestin therapy (oral MPA/megestrol) ± hysteroscopic resection; LNG-IUD ± systemic progestin; consider embryo/oocyte cryo if delay expected or advanced age.	After documented complete response on histologic reassessment (often 3–6-month intervals); early referral to REI; ART often encouraged to shorten time to pregnancy and reduce prolonged exposure to conservative management.	Higher recurrence risk; need scheduled hysteroscopic-guided biopsy surveillance; counsel on need for definitive hysterectomy after childbearing; implantation/receptivity issues can reduce live birth rates.
**Cervical** (early-stage SCC or HPV-related usual adenocarcinoma)	Fertility-sparing considered for tumor ≤2 cm, favorable histology (SCC/usual-type adeno), limited stromal invasion, no LVSI/limited risk features per guideline selection; imaging (MRI) and nodal assessment per protocol.	Conization (selected microinvasive) or trachelectomy (simple/radical; approach depends on size/risk); embryo/oocyte cryo if treatment may delay pregnancy or if stenosis risk expected.	After completion of fertility-sparing surgery and confirmation of oncologic control; ART can be used, especially if cervical stenosis/subfertility occurs.	Preterm birth, PPROM, second-trimester loss risk increased (especially after radical trachelectomy); often requires cesarean delivery (permanent cerclage); cervical stenosis may complicate conception/embryo transfer.
**Epithelial ovarian cancer** (selected early-stage)	Most appropriate for FIGO IA, low-grade (e.g., low-grade serous, mucinous expansile, endometrioid) with comprehensive staging; IC1 may be considered selectively with extensive counseling; generally not recommended for ≥IC2/IC3, grade 3, clear cell/high-risk biology, or ≥stage II.	Fertility-sparing surgery (unilateral salpingo-oophorectomy with staging; cystectomy only in select borderline cases/selected situations); consider embryo/oocyte cryo if adjuvant therapy planned or diminished reserve.	After recovery and oncologic clearance, individualized by stage/histology and adjuvant therapy; ART may be considered particularly if reserve reduced or unilateral ovary remaining.	Risk of recurrence depends on stage/histology; counsel on reduced ovarian reserve after surgery/chemotherapy; ART may improve conception in selected patients but timing should be individualized.
**BOT**, stage I	Common scenario for fertility-sparing; stage I BOT typically eligible; bilateral disease and micropapillary/implant features require individualized counseling.	Cystectomy or unilateral oophorectomy depending on laterality/recurrence risk; embryo/oocyte cryopreservation if high recurrence risk or anticipated repeat surgery.	After surgery once stable; ART can be considered and may increase pregnancy rates in some series; coordinate surveillance.	Recurrence risk higher with cystectomy; long-term follow-up; pregnancy generally feasible; counsel on repeat surgery possibility.

Abbreviations: FP: fertility preservation; ART: assisted reproductive technology; HR: hormone receptor; GnRH: gonadotropin releasing hormone; REI: reproductive endocrinology and infertility; AH: atypical hyperplasia; EIN: endometrial intraepithelial neoplasia; EC: endometrial cancer (invasive); MRI: magnetic resonance imaging: TVUS: transvaginal ultrasound; SCC: squamous cell carcinoma; HPV: human papilloma virus; LVSI: lymphovascular space invasion; LNG-IUD: levonorgestrel intrauterine device; PPROM: preterm prelabor rupture of membranes; FIGO: International Federation of Gynecology and Obstetrics.

**Table 2:** ART use and oncologic outcomes after cancer treatment.

Study (Year)	Study Design	N	Cancer Type/Tage	ART/FP Exposure	Comparator	Oncologic Outcome	Pregnancy/ Livebirth/ Recurrence Rates	Key Findings/ Limitations
Azim et al. (2024) [67]	Prospective, single-arm	518	Stage I-III HR-positive breast cancer	ET interruption ± prior FP/ART	Internal comparison	BCFI/recurrence	74%/NR/8.7–9.7%	No short-term recurrence signal at 41 mo
Wang et al. (2022) [65]	Retrospective	153	Stage I-III breast cancer	FP ± ART	No FP	Recurrence	40–66%/30–52%/13.4–14.1%	No increase in recurrence
Novikova et al. (2021) [70]	Retrospective	418	AEH and grade 1–2 endometrioid EC with no or minimal myometrial invasion	ART after CR	No ART	Recurrence	NR/42%/26–36%	No excess recurrence
Nezhat et al. (2020) [71]	Systematic review	3044	Stage IA1-IB1 cervical cancer	ART post-trachelectomy	-	Recurrence	55.4%/67.9%/3.2%	No oncologic compromise
Daraï et al. (2013) [72]	Systematic review	1694 ^1^	BOT	ART after FSS	-	Recurrence	54% vs. 80% (spontaneous vs. ART)/NR/0–25%	ART feasible in selected pts

Abbreviations: N: sample size; FP: fertility preservation; ART: assisted reproductive technology; HR: hormone receptor; ET: endocrine therapy; BCFI: breast cancer free interval; NR: Not reported; AEH: atypical endometrial hyperplasia; EC: endometrial carcinoma; CR: clinical response; BOT: Borderline ovarian tumor; FSS: fertility-sparing surgery. ^1^ Only series including >50 cases.

**Table 3:** Key obstetric outcomes after fertility-sparing cancer treatment.

Cancer Type	Fertility-Sparing Treatment	Conception Mode	Live Birth Rate	Preterm Birth Risk	Delivery Considerations
**Cervical**	Simple trachelectomy	Spontaneous/ART	65%	86%	Often CS
**Cervical**	Radical trachelectomy	ART common	42–68%	42–64%	CS mandatory
**Endometrial**	Progestin ± LNG-IUD	ART encouraged	18–61%	13–45%	Standard
**Ovarian (FSS)**	USO/cystectomy	ART optional	23–79%	20–73%	Standard

Abbreviations: ART: assisted reproductive technology; CS: cesarean section; FSS: fertility-sparing surgery; USO: unilateral salpingo-oophorectomy.

## Data Availability

No new data were created or analyzed in this study. Data sharing is not applicable to this article.
